# A New Type of Wireless Transmission Based on Digital Direct Modulation for Use in Partially Implantable Hearing Aids

**DOI:** 10.3390/s21082809

**Published:** 2021-04-16

**Authors:** Jong-Hoon Kim, Jin-Ho Cho, Ki-Woong Seong, Myoung-Nam Kim

**Affiliations:** 1 Institute of Biomedical Engineering Research, Kyungpook National University, 680 Gukchaebosang-ro, Jung-gu, Daegu 41944, Korea; jh850526@naver.com (J.-H.K.); jhcho@ee.knu.ac.kr (J.-H.C.); 2 Department of Biomedical Engineering, Kyungpook National University Hospital, 130 Dongdeok-ro, Jung-gu, Daegu 41944, Korea; 3 Department of Biomedical Engineering, School of Medicine, Kyungpook National University, 680 Gukchaebosang-ro, Jung-gu, Daegu 41944, Korea

**Keywords:** partially implantable hearing aids, inductive coupling, wireless transmission system, low distortion, sigma-delta modulation

## Abstract

In this study, we developed a new type of wireless transmission system for use in partially implantable hearing aids. This system was designed for miniaturization and low distortion, and features direct digital modulation. The sigma-delta output, which has a high SNR due to oversampling and noise shaping technology, is used as the data signal and is transmitted using a wireless transmission system to the implant unit through OOK without restoration as an audio signal, thus eliminating the need for additional circuits (i.e., LPF and a reference voltage supply circuit) and improving the ease of implantation and reliability of the circuit. We selected a carrier frequency of 27 MHz after analysis of carrier attenuation by human tissue, and designed the communication coil with reference to both the geometry and required communication distance. Circuit design and simulation for wireless transmission were performed using Multisim 13.0. The system was fabricated based on the circuit design; the size of the device board was 13 mm × 13 mm, the size of the implanted part was 9 mm × 9 mm, the diameter of the transmitting/receiving coil was 26 mm, and the thicknesses of these coils were 0.5 and 0.3 mm, respectively. The difference (error) between the detected and simulation waveforms was about 5%, and was thought to be due to the tolerances of the fabricated communication coil and elements (resistors, capacitors, etc.) used in the circuit configuration of the system. The number of windings was reduced more than 9-fold compared to the communication coil described by Taghavi et al. The measured THD was <1% in the frequency band from 100 Hz to 10 kHz, thus easily meeting the standard specification for hearing aids.

## 1. Introduction

Due to the aging of the population and frequent use of multimedia devices, the incidence of hearing loss is increasing in various age groups [1,2,3,4]. There is increasing interest in the use of hearing aids to overcome the problems associated with hearing loss, which include difficulties in communication, learning disabilities, and reduced cognitive ability [5]. Although air conduction hearing aids are widely used for hearing compensation, they have a number of drawbacks, such as occlusion effects, howling noise, and pain [6]. Moreover, the adoption rate of conventional hearing aids remains quite low due to concerns about people’s negative perspectives from external exposure [7,8]. Various types of implantable hearing aids are being actively studied to address these issues [9,10,11,12,13,14,15]. Implantable hearing aids can be divided into fully and partially implantable types. Fully implantable devices have cosmetic benefits, but leakage of the built-in rechargeable battery fluid may occur. In addition, they have not been commercialized because the surgery required for installation is complicated and a further surgical procedure is necessary for battery replacement [16]. Partially implantable hearing aids are divided into external device and implant unit. Since the implant unit does not have a built-in rechargeable battery, there is no problem about battery fluid leakage and surgery for battery replacement. Therefore, partially implantable hearing aids are widely used due to the minimal associated risks and simple installation surgery [17].

The Rion implantable hearing aid system developed by Yanagihara et al. uses a baseband transmission method that wirelessly transmits audio signals without a modulation process. After amplification, the audio signal is transmitted to the implant unit without modulation, and the transmitted signals directly drive the transducer [18]. However, there are several problems associated with the baseband transmission method. First, as the parasitic capacitance of the communication coil is very small, of the order of a few pF, it has a self-resonant frequency of several hundred MHz. Here, the self-resonant frequency of the communication coil is inversely proportional to the inductance and parasitic capacitance of the coil; to locate the self-resonant frequency in the audio signal region (low-frequency region), the number of windings of the coil must be high. In addition, even with an LC resonance network in a low-frequency band, the low-frequency characteristics will be poor because the resonance frequency is not flat. Therefore, as the low-frequency gain is low, these devices are only applicable for hearing compensation in patients experiencing the loss of high-frequency sound perception. Second, as the amplitude of the signal is susceptible to ambient interference, baseband transmission is affected by noise during transmission, and signal distortion occurs. Therefore, a process for converting the audio signal into a specific frequency is required. Recently, Taghavi et al. developed a system featuring amplitude modulation (AM), which transforms the sigma-delta output of a digital signal processor (DSP) chip into an audio signal via a low-pass filter (LPF), after which the signal is transmitted to the implant unit [19,20]. However, the system is large due to inclusion of the LPF to convert the sigma-delta output into an audio signal and the circuit for supplying the reference voltage. In addition, the power efficiency of the output stage using a class-A amplifier is low. Also, the communication coil is large given the low carrier frequency (120 kHz). The system is very vulnerable to noise because it performs AM based on the audio signal. Therefore, there is a need to develop smaller systems with low distortion.

In this paper, we describe a new type of wireless transmission system for use in partially implantable hearing aids developed with the aim of miniaturization and low distortion, and featuring direct digital modulation. The sigma-delta output, which has a high SNR due to oversampling and noise shaping technology, is used as the data signal and is transmitted using a wireless transmission system to the implant unit through OOK without restoration as an audio signal, thus eliminating the need for additional circuits. In order to select a carrier frequency, we considered the characteristics of the sigma-delta output, the anatomical needs of the patient, and the attenuation behaviors of electromagnetic waves. Circuit design and simulation for wireless transmission were performed using Multisim 13.0, and the system was fabricated based on the circuit design. Finally, performance was verified by measuring the total harmonic distortion (THD) by reference to the acceptable hearing aid specifications.

## 2. Design Considerations

### 2.1. Conventional Partially Implantable Hearing Aids

**Figure 1:**
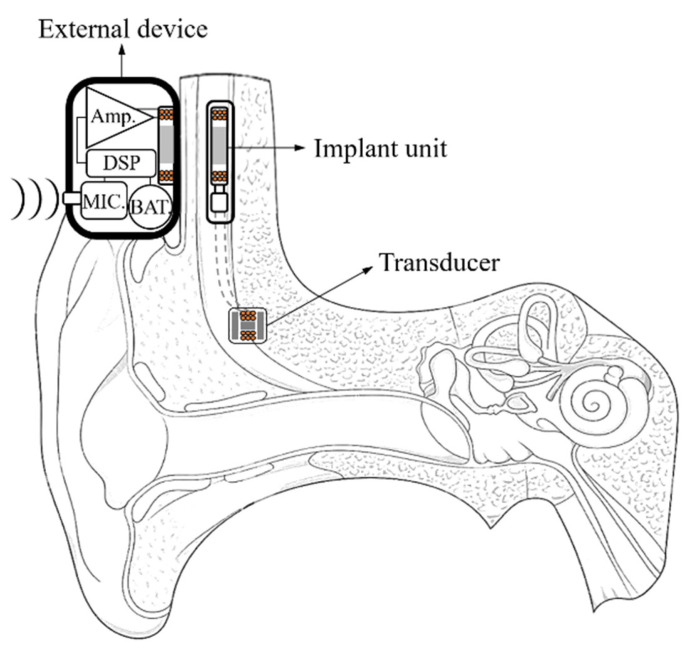
The conceptual diagram of the partially implantable hearing aid.

The partially implantable hearing aid consists of an external device and an implant unit; the conceptual diagram is shown in Figure 1. The principle of a partially implantable hearing aid is classified as several steps. First, the audio signal is converted into an electrical signal through a microphone. Second, the converted electrical signal is output as a digital signal through DSP. Third, the digital signal is converted as an analog signal through a filter. Fourth, the analog signal is modulated to carrier frequency and transmitted to the implant unit through inductive coupling. Finally, the modulation signal that is transmitted to implant unit is restored to an audio signal through a demodulation circuit; it is transmitted to the transducer [21].

In general, most hearing aid chips have a built-in DSP, and in order to analog to digital convert (ADC) of low noise and high resolution, a sigma-delta ADC is used. However, the conventional partially implantable hearing aids restore the sigma-delta output of DSP to an analog signal, and it is transmitted to the implant unit. In this case, there is no advantage of the sigma-delta output and it is easy to be exposed to noise. In this paper, a new wireless transmission system that makes use of the sigma-delta output was designed.

### 2.2. Sigma-Delta Output Characteristics

**Figure 2:**
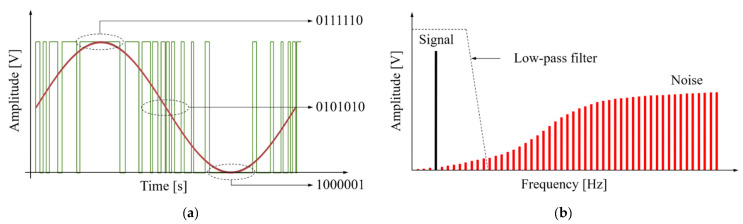
The sigma-delta output: (**a**) Time domain; (**b**) Frequency domain.

The sigma-delta converter is a digital modulation method that increases signal-to-noise ratio (SNR) by using oversampling and noise shaping techniques [22]. The time and frequency domain of sigma-delta output is shown in Figure 2. As shown in Figure 2a, the sigma-delta output is a signal that represents the amplitude of the audio signal as the difference in the pulse density. In other words, the relative density of the pulses corresponds to the amplitude of the audio signal because these sigma-delta output uses only 1-bit to record the signal at any sampling point. Then, as shown in Figure 2b, the sigma-delta output can be restored to an audio signal using LPF because it contains information of the original signal in the low frequency domain [23]. If the sigma-delta output is used for wireless transmission without restoring an audio signal, not only is the distortion low, but also the volume of the system is reduced by simplifying the circuit configuration. However, when the sigma-delta output is transmitted into the implant unit via inductive coupling, the problem occurs. If the communication coil is designed for the sigma-delta output that is oversampled from several hundreds of kHz to several MHz, the audio signal placed in the low frequency band is hardly transmitted. In other words, transmission efficiency is poor. Therefore, in order to transmit the sigma-delta output with high efficiency, a modulation process is needed that can transmit an audio signal of the low-frequency band in the spectrum of the sigma-delta output.

### 2.3. Proposed Wireless Transmission System

**Figure 3:**
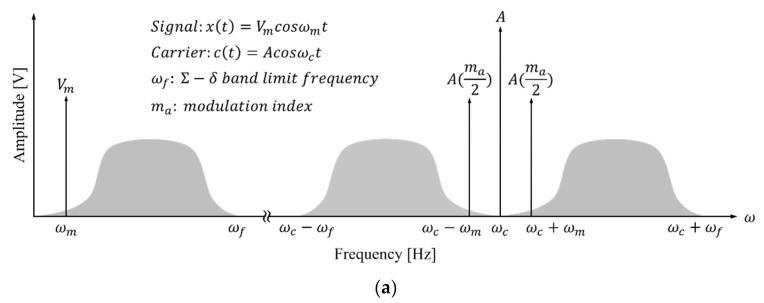

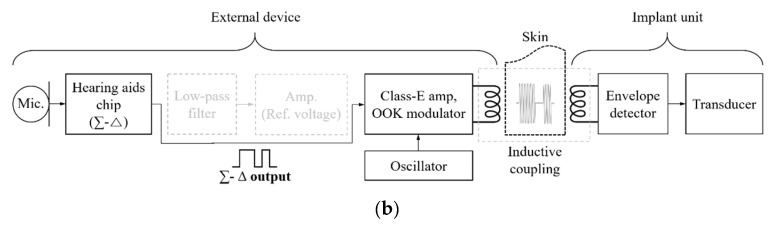
(**a**) Frequency domain of modulated sigma-delta output; (**b**) structure of proposed wireless transmission system.

In this paper, we proposed a wireless transmission method using on-off keying (OOK) modulation to transmit an audio signal placed in the low-frequency band of the sigma-delta output. The operating principle of the proposed system can be divided into several stages. First, as shown in Figure 3a, the sigma-delta output band-limited to $\omega_{f}$ is modulated to the carrier frequency ($\omega_{c}$) using OOK. Second, the modulated sigma-delta output is located in the $\omega_{c}\pm \omega_{m}$ band. Third, the modulation signal is supplied to the communication coil through a class-E amplifier. Finally, the modulation signal transmitted to the implant unit is restored to a sigma-delta output through an envelope detector. Noise is still present in the restored sigma-delta output, but can be effectively removed with the LPF.

Figure 3b shows the proposed wireless transmission system that can achieve device miniaturization and low distortion. The external device of the proposed system consists of a microphone, DSP, a tuned class-E switching power amplifier including AM, and a transmitting coil. This external device delivers amplitude-modulated sigma-delta output to the implant unit. The implant unit consists of a receiving coil, an envelope detector, and transducer; it receives the amplitude-modulated sigma-delta output via skin. Unlike conventional methods, the distortion is low because the sigma-delta output is used directly, and the volume of the proposed system is reduced because the sigma-delta output is not restored to the audio signal.

### 2.4. Carrier Frequency Selection

As the sigma-delta output is oversampled, a high carrier frequency is required for wireless transmission; this allows miniaturization of the communication coil. However, if the carrier frequency is too high, attenuation is generated by human tissue during wireless transmission. Therefore, the carrier frequency should be selected by reference to the skin thickness. In general, the implant unit of a partially implantable device lies under the temporal scalp, the thickness of which is 7 mm (2 mm of skin, 1 mm of fat, and 4 mm of muscle) [24,25]. The communication distance is, thus, 9 mm when the thicknesses of the device case and the silicon coating of the receiving coil are added.

The frequency bands used in implantable medical devices include the Industrial Scientific Medical (ISM) and Medical Implant Communication Services (MICS) bands. We selected four frequencies (13.56, 27, and 402 MHz; and 2.4 GHz) from the ISM and MICS bands for attenuation analysis. The transmission depth of the carrier wave is that of the temporal scalp thickness; all attenuation analyses assumed the skin, fat, and muscle thicknesses given above. According to the Beer–Lambert law, the carrier wave strength decreases as the transmission depth increases; such attenuation is expressed by Equation (1) [26]:

(1) $$W_{d}=W_{t}e^{\left(-2\beta d\right)}$$

where $W_{d}$ is the carrier wave energy flux density at the transmission depth, $W_{t}$ is the incident energy density of the carrier wave, d is the transmission depth, and β is the attenuation coefficient of tissue. The transmission depth *d* is 7 mm, and β is calculated by Equation (2):

(2) $$\beta =\;\omega {\left[\left(\frac{\mu \varepsilon }{2}\right)\left(\sqrt{1+\frac{\sigma^{2}}{\varepsilon^{2}\omega^{2}}}-1\right)\right]}^{1/2}$$

where *σ* is the conductivity of tissue, *μ* is the permeability of tissue, *ε* is the relative permittivity of tissue, and *ω* is the carrier frequency. As shown in Equation (2), the attenuation coefficient of tissue increases as the carrier frequency increases, and depends on the electrical properties of the tissue. *μ* is regarded as a constant because it is not frequency-dependent [27]. As *σ* and *ε* are frequency-dependent, the attenuation coefficients were deduced for the four frequencies, as shown in Table 1 [28]. The attenuation of the carrier wave was analyzed based on the *σ* and *ε* values of skin, fat, and muscle (Table 2). As shown in Table 2, at carrier frequencies with attenuation rates less than 5% (13.56 and 27 MHz), the attenuation rate difference was 0.3%. This is very small and can be ignored. Moreover, the higher the carrier frequency, the lower the ripple rate of the envelope detector. In other words, efficient detection is possible. Therefore, we selected a carrier frequency of 27 MHz given the efficiency of envelope detection; this selection is verified in Section 3.

## 3. System Design and Fabrication

### 3.1. Communication Coil Design

**Figure 4:**
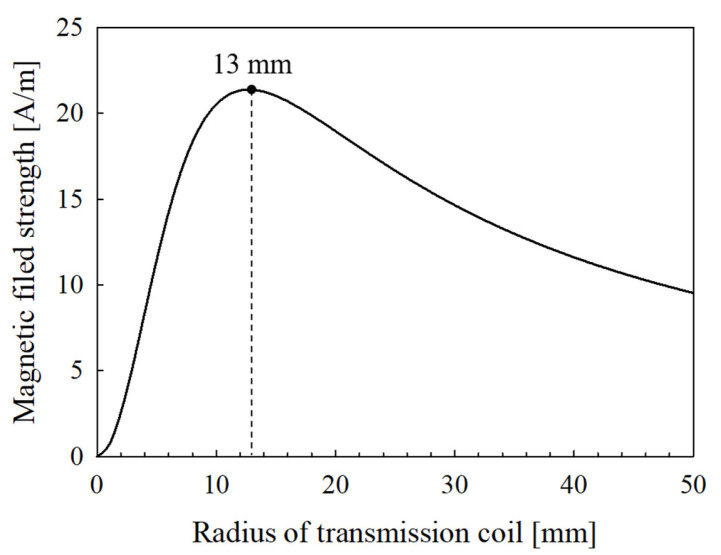
Intensity of magnetic field according to radius of the transmitting coil at the communication distance 9 mm.

The wireless transmission system features inductive coupling; the communication distance was 9 mm (please see above). To meet these conditions, the analysis of the magnetic flux generated by the radius of the transmitting coil is performed based on communication distance, and which is calculated by Equation (3) [29]:

(3) $$H=\frac{INR_{t}^{2}}{2\sqrt{{\left(R_{t}+z^{2}\right)}^{3}}}$$

where H is the magnetic field strength, $R_{t}$ is the radius of the transmitting coil, z is the communication distance, I is the electric current, and N is the number of windings. The I and N are constants, respectively, and H and $R_{t}$ are variables. When H is the maximum, $R_{t}$ is optimized, and the results are shown in Figure 4.

As shown in Figure 4, $R_{t}$ is determined to be 13 mm. The inductance of the transmitting coil corresponds to that of the LC resonance network of the Class-E amplifier, thus 1.46 µH using the formula of Sokal [30]. The transmitting coil is designed based on the deduced $R_{t}$ and L values; a spiral coil was chosen to minimize lateral misalignment. N can be derived from the inner diameter of the coil and the diameter of the copper wire, as shown in Equations (4) and (5) [31]:

(4) $$N=\sqrt{\frac{L\left(30T-11D_{min}\right)}{T^{2}}}$$

(5) $$T=\frac{D_{min}+\left(NW_{D}\right)}{2}$$

where L is the inductance, $D_{min}$ is the inner diameter of the coil, $W_{D}$ is the diameter of the copper wire, and T is the variable that allows coil inductance to be derived. $W_{D}$ is a constant, and L is derived by changing N and $D_{min}$. The results are shown in Table 3; the optimal N was 6. In addition, if the coils are of the same size, the coupling coefficient is higher than when the coil sizes differed. Therefore, the radius of the spiral receiving coil was 13 mm, equivalent to that of the transmitting coil.

### 3.2. Circuit Design and Smulation

**Figure 5:**
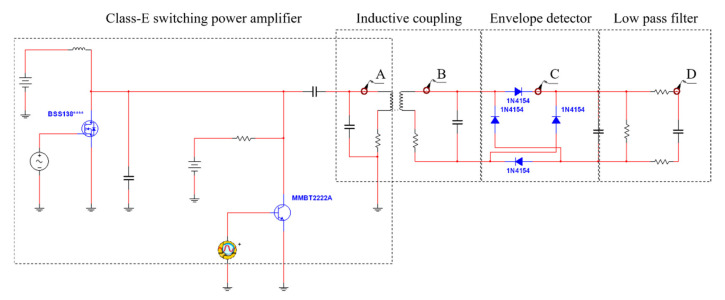
The designed wireless transmission system using Multisim.

Circuit design was performed with the aid of Multisim ver. 13.0 (National Instruments, Austin, TX, USA); the circuit is shown in Figure 5 and includes an AM component, a class E power amplifier, inductive coupling, and envelope detector. We considered all of the communication coil parameters and parasitic components. The sigma-delta output of the SA3291 (ON Semiconductor, Phoenix, AZ, USA) was extracted using DIAdem software (National Instruments, Austin, TX, USA) and used in simulation.

**Figure 6:**
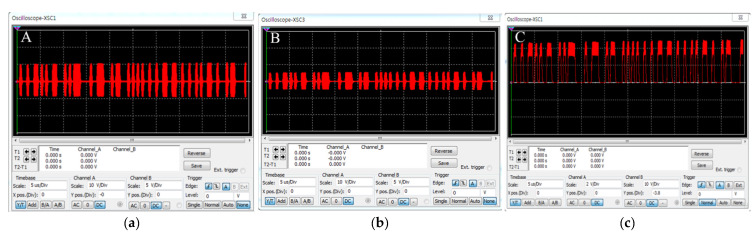
Simulation waveform of the designed wireless transmission system: (**a**) Transmitting coil; (**b**) Receiver coil; (**c**) Envelope detector.

**Figure 7:**
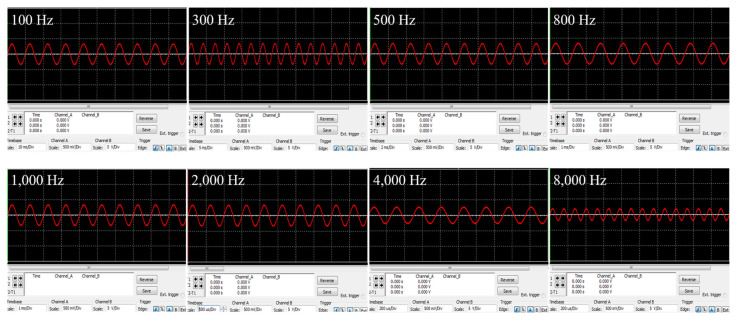
Simulation waveforms restored by a low-pass filter.

In order to verify operation of the proposed wireless transmission system, the simulation of the designed circuit is performed, and the output waveforms for each circuit are shown in Figure 6. As shown in Figure 6, the carrier (frequency 27 MHz) was modulated by the sigma-delta output that switched the MOSFET, and the modulated signal of 19.6 V was output through the LC resonance network of the class E power amplifier. The modulated signal was wirelessly transmitted via inductive coupling; the output voltage of the receiving coil was 10.3 V. The transmitted signal was detected as a sigma-delta output by the envelope detector. This output was restored to an audio signal via the LPF; the restored signal from 100 to 8000 Hz is shown in Figure 7.

**Figure 8:**
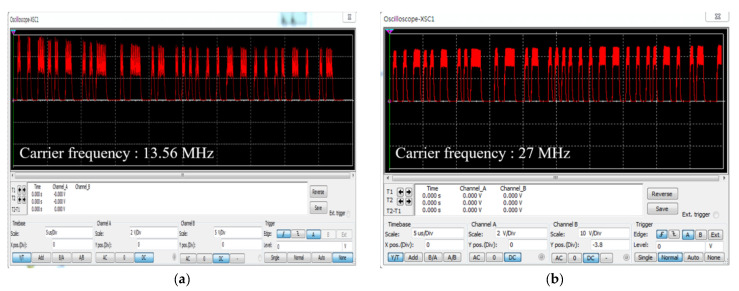
The output signal detected by the envelope detector: (**a**) 13.56 MHz carrier frequency; (**b**) 27 MHz carrier frequency.

To verify the validity of our carrier frequency selection, we performed further simulations; the results are shown in Figure 8. The demodulation efficiencies of signals modulated at 13.56 and 27 MHz and then transmitted to the implant were analyzed. The demodulation efficiency is the ripple factor ($R_{f}$) of the detected signal as given by Equation (6):

(6) $$R_{f}=\frac{V_{max}-V_{min}}{V_{DC}}100$$

where $V_{min}$ is the minimum value of the ripple voltage, $V_{max}$ is the maximum value of that voltage, and $V_{DC}$ is the average value of that voltage. The 13.56 and 27 MHz comparisons were based on Equations (1) and (6), and Figure 8; the results are shown in Table 4. We confirmed that the attenuation factor at 27 MHz was 0.3% higher than that at 13.56 MHz, but a voltage gain of 0.6 V was confirmed at the $V_{DC}$. As an attenuation factor of 0.3% is very small compared to such a voltage gain, the validity of our carrier frequency selection was confirmed.

### 3.3. System Fabrication

**Figure 9:**
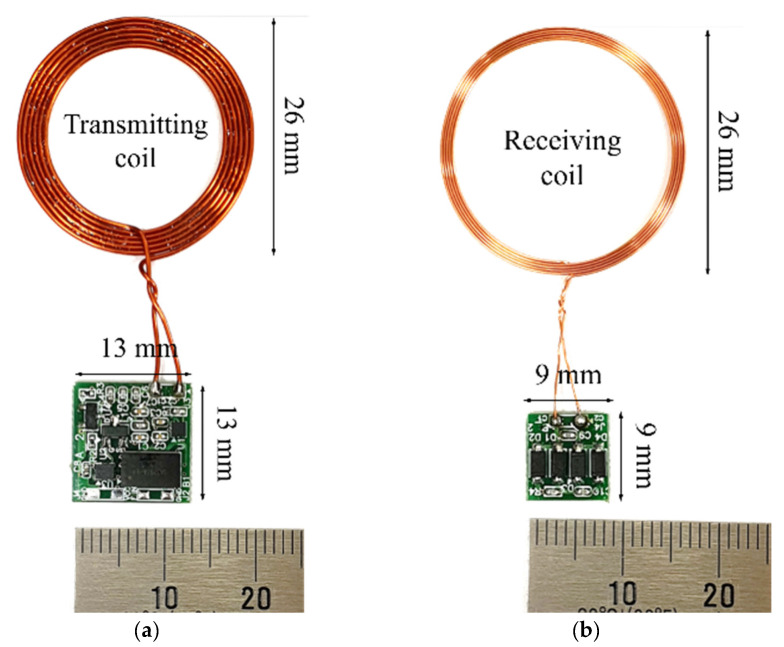
Fabricated wireless transmission system: (**a**) External device board; (**b**) Implant board.

**Figure 10:**
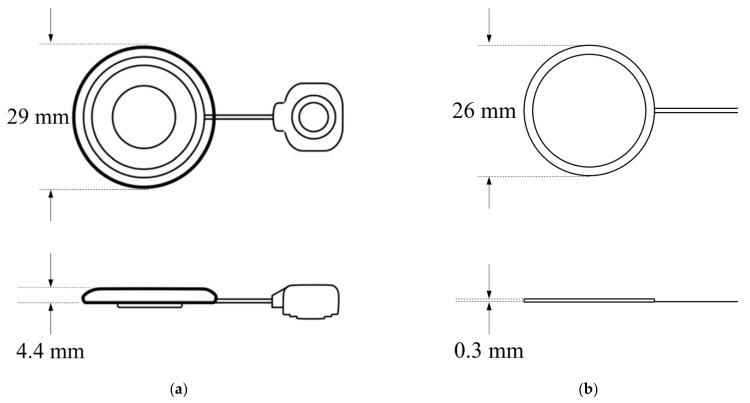
(**a**) Receiver coil (Taghavi et al.) [32]; (**b**) fabricated receiver coil.

The proposed wireless transmission system was fabricated based on the design and simulation discussed in the previous section, and the printed circuit board and communication coil of the fabricated system are shown in Figure 9. The receiving coil described by Taghavi et al., and the proposed receiving coil, are shown in Figure 10 [32]. As shown in the figure, the thickness of the proposed coil is significantly reduced; however, accurate comparison is difficult because only the dimensions of the silicon-coated receiving coil are shown. To verify that miniaturization was feasible, the number of windings in the coil described by Taghavi et al. was deduced based on the inductance value (150 *u*H) [20] using Equations (4) and (5). We compared our coil with that described by Taghavi et al. based on the deduced results, and the number of windings was shown to differ by about 9-fold (Table 5). Increasing the inner diameter to reduce the number of windings increases the diameter of the coil. Conversely, if the inner diameter is decreased, the thickness of the coil increases because more windings are required. We confirmed that our coil was smaller in diameter and thinner than that described by Taghavi et al.

## 4. Results

### 4.1. Operational Verification

**Figure 11:**
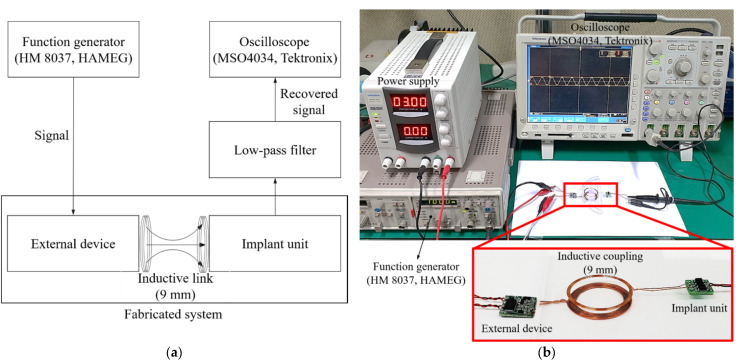
Operation experiment of the fabricated total wireless transmission system: (**a**) Block diagram; (**b**) Experimental environment.

**Figure 12:**
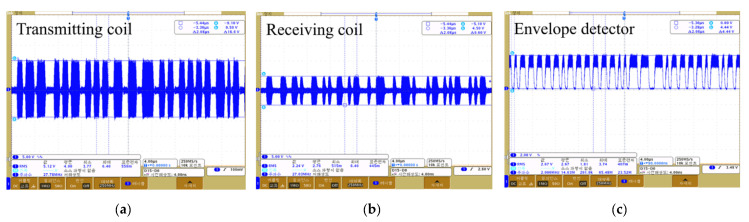
Output waveform of the fabricated wireless transmission system: (**a**) Transmitting coil; (**b**) Receiving coil; (**c**) Envelope detector.

**Figure 13:**
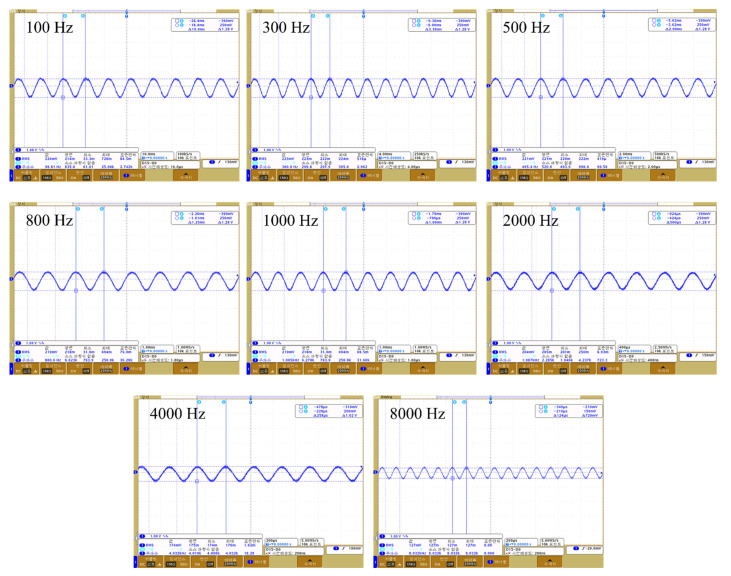
Analog signal restored by a low-pass filter.

We experimentally verified the appropriate operation of our wireless transmission system; the block diagram and experimental environment are shown in Figure 11a,b. A signal from 100 to 8000 Hz was applied to the external device, and the output waveform of each circuit measured using an oscilloscope. A dummy load of 220 ohm served as the system load; the communication distance was 9 mm. The measured waveforms are shown in Figure 12. Figure 12a,b are the waveforms measured at the transmitting and receiving coils respectively; voltages of 18.6 and 9.6 V were output. Figure 12c is the waveform measured by the envelope detector; this was a sigma-delta output of 4.44 V. The sigma-delta output was recovered as an audio signal via the LPF, and the signal recovered from 100 to 8000 Hz is shown in Figure 13.

Comparison of the detected and simulation waveforms indicated a difference (error) of about 5%, which was attributed to the tolerance of the fabricated communication coil and elements (resistors, capacitors, etc.) used in the circuit configuration of the system. Overall, the experimental and simulation results tended to be similar. As shown in Figure 12c, ripples occurred in the sigma-delta output during the demodulation process. The relative density of the pulses corresponded to the amplitude of the audio signal, because the sigma-delta output uses only 1 bit to record the signal at any sampling point. That is, the generated ripples are not perceived as noise because the information of the audio signal exists within the pulse width of the sigma-delta output. In addition, as the generated ripples have a frequency higher than the audible range, distortion will not be increased. Based on the experiments, when a dummy load of 200 Ω was applied at a communication distance of 9 mm, power transmission of 7.4 mW was achieved. The transmitted power should be sufficient to drive the transducer, and a transducer design taking the performance of the wireless transmission system into consideration will be required in future.

### 4.2. Total Harmonic Distortion

**Figure 14:**
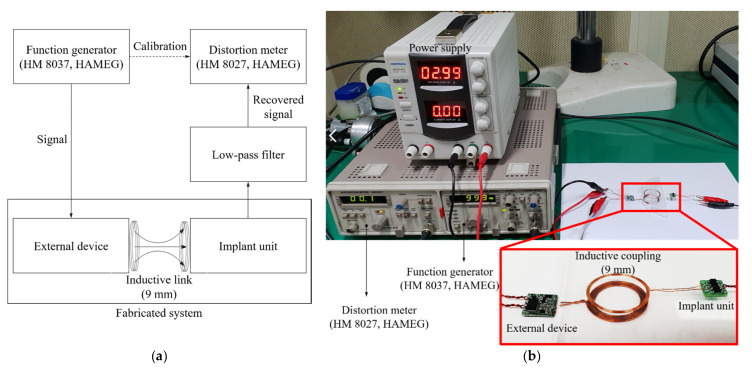
Total harmonic distortion measurement: (**a**) Experimental environment; (**b**) Measurement result of total harmonic distortion.

Total harmonic distortion (THD) affects the performance of partially implantable hearing aids. It is necessary to take the THD of the wireless transmission system into consideration for clear sound transmission to the implant unit. To verify the fabricated wireless transmission system, we evaluated the THD with reference to the standard hearing aid specification (ANSI S3.22-2003) [33]. The block diagram and experimental environment are shown in Figure 14a,b, respectively.

**Figure 15:**
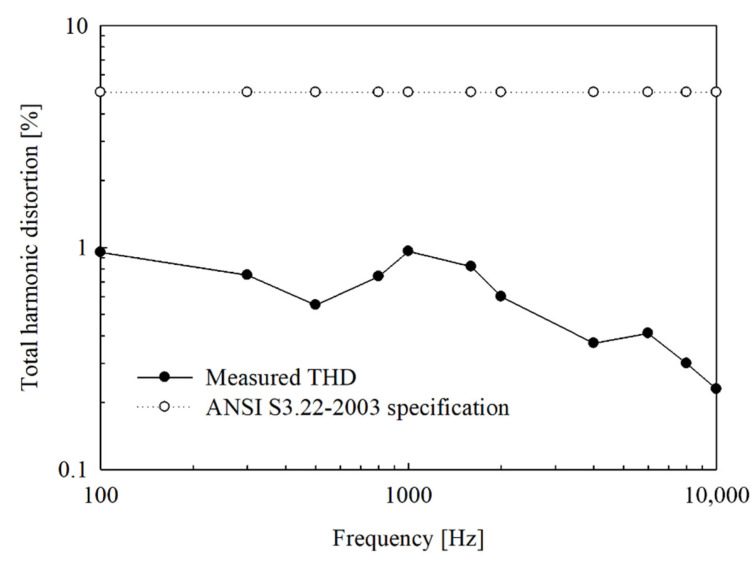
Measurement result of total harmonic distortion.

First, the signal is applied from the signal generator to the distortion meter, and distortion is corrected at the applied signal frequency (reference signal for distortion measurement). Second, the signal is applied from the signal generator to the wireless transmission system; the applied signal is output as a sigma-delta signal through DSP and then modulated. Third, the modulated sigma-delta output is transmitted to the implant unit via inductive coupling. Fourth, the transmitted sigma-delta output is restored to the original signal through a low-pass filter. Finally, the reconstructed signal is applied to a distortion meter to measure the THD. In Figure 15, the upper curve shows the THD required when using the standard specification of the hearing aid, and the lower curve shows the THD measured in the fabricated wireless transmission system. The measured THD was <1% in the frequency band from 100 Hz to 10 kHz, thus easily meeting the standard specification.

## 5. Discussion and Conclusions

In this study, we developed a new type of wireless transmission system for use in partially implantable hearing aids. This system was designed for miniaturization and low distortion, and features direct digital modulation. The sigma-delta output, which has high SNR due to oversampling and noise shaping technology, is used as the data signal and transmitted using a wireless transmission system to the implant unit through OOK without restoration as an audio signal, thus eliminating the need for additional circuits (i.e., LPF and a reference voltage supply circuit) and improving the ease of implantation and reliability of the circuit. We selected a carrier frequency of 27 MHz after analysis of carrier attenuation by human tissue, and designed the communication coil with reference to both the geometry and required communication distance. Circuit design and simulation for wireless transmission were performed using Multisim 13.0. The system was fabricated based on the circuit design; the size of the device board was 13 mm × 13 mm, the size of the implanted part was 9 mm × 9 mm, the diameter of the transmitting/receiving coil was 26 mm, and the thicknesses of these coils were 0.5 and 0.3 mm, respectively. The difference (error) between the detected and simulation waveforms was about 5%, and was thought to be due to the tolerances of the fabricated communication coil and elements (resistors, capacitors, etc.) used in the circuit configuration of the system. The number of windings was reduced more than 9-fold compared to the communication coil described by Taghavi et al. The measured THD was < 1% from 100 Hz to 10 kHz, thus easily meeting the standard specification for hearing aids. Based on the experiments, when a dummy load of 200 Ω was applied at a communication distance of 9 mm, power transmission of 7.4 mW was achieved. Therefore, the results of this study should aid the development of a partially implanted hearing aid, and the advantages of circuit miniaturization and low distortion are expected to be beneficial for wearable devices and bio-health applications. However, the current consumption is 60 mA with a 3 V supply, and further research is required to reduce the consumption to allow the design of a practical circuit.

## Figures and Tables

**Table 1:** Conductivity and permittivity of the skin, muscle, and fat in ISM and MICS bands [28].

		Skin	Fat	Muscle
13.56 MHz	Conductivity, *σ*	0.23	0.055	0.64
	Permittivity, *ε*	295.24	25.32	140.40
27 MHz	Conductivity, *σ*	0.30	0.06	0.65
	Permittivity, *ε*	191.99	18.76	98.93
402 MHz	Conductivity, *σ*	0.69	0.08	0.81
	Permittivity, *ε*	46.79	11.66	53.60
2,400 MHz	Conductivity, *σ*	1.49	0.28	1.69
	Permittivity, *ε*	38.54	10.93	53.25

**Table 2:** Attenuation ratio by the human tissue in ISM and MICS bands (derived by Equations (1) and (2) and Table 1).

**Frequency [MHz]**	**Attenuation Ratio [%]**
13.56	4
27	4.3
402	10.1
2400	21.1

**Frequency [MHz]**

**Attenuation Ratio [%]**

**Table 3:** Inductance value according to the number of windings.

$D_{min}[\mathrm{mm}]$	$W_{D}[\mathrm{mm}]$	N	L [µH]
22	0.5	4	0.76
21	0.5	5	1.10
20	0.5	6	1.48
19	0.5	7	1.90

**Table 4:** Comparison of ripple factor, ripple voltage, and attenuation ratio at 13.56 and 27 MHz.

	13.56 MHz	27 MHz
Ripple factor [%]	58.8	20
DC portion [V]	3.4	4
Attenuation ratio [%]	4	4.3

**Table 5:** Size comparison between receiver coil (Taghavi et al.) and fabricated receiver coil.

	Receiver Coil (Taghavi et al.)	Fabricated Receiver Coil
Diameter of the copper wire [mm]	0.3	0.3
Inner diameter of the coil [mm]	22.4	22.4
Inductance [µH]	149.31	1.77
Number of windings	58	6

## Data Availability

Not applicable.
